# Fine-mapping of a QTL influencing pork tenderness on porcine chromosome 2

**DOI:** 10.1186/1471-2156-8-69

**Published:** 2007-10-12

**Authors:** Stacey N Meyers, Sandra L Rodriguez-Zas, Jonathan E Beever

**Affiliations:** 1 Department of Animal Sciences, University of Illinois at Urbana-Champaign, Urbana, IL 61801, USA

## Abstract

**Background:**

In a previous study, a quantitative trait locus (QTL) exhibiting large effects on both Instron shear force and taste panel tenderness was detected within the Illinois Meat Quality Pedigree (IMQP). This QTL mapped to the q arm of porcine chromosome 2 (SSC2q). Comparative analysis of SSC2q indicates that it is orthologous to a segment of human chromosome 5 (HSA5) containing a strong positional candidate gene, calpastatin (*CAST*). *CAST *polymorphisms have recently been shown to be associated with meat quality characteristics; however, the possible involvement of other genes and/or molecular variation in this region cannot be excluded, thus requiring fine-mapping of the QTL.

**Results:**

Recent advances in porcine genome resources, including high-resolution radiation hybrid and bacterial artificial chromosome (BAC) physical maps, were utilized for development of novel informative markers. Marker density in the ~30-Mb region surrounding the most likely QTL position was increased by addition of eighteen new microsatellite markers, including nine publicly-available and nine novel markers. Two newly-developed markers were derived from a porcine BAC clone containing the *CAST *gene. Refinement of the QTL position was achieved through linkage and haplotype analyses. Within-family linkage analyses revealed at least two families segregating for a highly-significant QTL in strong positional agreement with *CAST *markers. A combined analysis of these two families yielded QTL intervals of 36 cM and 7 cM for Instron shear force and taste panel tenderness, respectively, while haplotype analyses suggested further refinement to a 1.8 cM interval containing *CAST *markers. The presence of additional tenderness QTL on SSC2q was also suggested.

**Conclusion:**

These results reinforce *CAST *as a strong positional candidate. Further analysis of *CAST *molecular variation within the IMQP F_1 _boars should enhance understanding of the molecular basis of pork tenderness, and thus allow for genetic improvement of pork products. Furthermore, additional resources have been generated for the targeted investigation of other putative QTL on SSC2q, which may lead to further advancements in pork quality.

## Background

A major objective of the swine industry is to supply high-quality, nutritious pork for consumers. To meet consumer demand, it is necessary for animal producers to recognize and understand both the genetic and environmental factors influencing pork quality. The genetic component of meat quality is complex, i.e. many economically important quality traits, such as color, flavor, juiciness, fat content and tenderness, are controlled by several genes throughout the genome referred to as quantitative trait loci (QTL).

Recently, a QTL with large effects on pork tenderness was detected within the Illinois Meat Quality Pedigree (IMQP), composed of 832 F_2 _individuals originating from a Berkshire × Duroc intercross [1]. QTL exceeding the genome-wise significance threshold of p < 0.0001 were detected, at essentially the same position on porcine chromosome 2 (SSC2), for both Instron shear force and taste panel tenderness (the mechanical and sensory measurements of tenderness, respectively). However, due to the small number of markers used in the linkage analysis, this QTL has been vaguely positioned within a large marker interval of approximately 60 centimorgan (cM). This interval is bounded by markers SW1026, located at the centromeric end of SSC2p, and SW1844, located at the telomeric end of SSC2q [2-4]. Thus, the marker interval for this QTL includes almost the entire q arm of SSC2. This region is too large to effectively interrogate and must be refined to facilitate positional cloning of the responsible gene.

The largest comparative segment of SSC2q, spanning more than half of this chromosome arm, is orthologous to an approximately 128-Mb region of human chromosome 5 (HSA 5) [3]. Among the genes in this region is a single obvious candidate – calpastatin (*CAST*). Calpastatin is a specific inhibitor of some calcium-dependent proteases, known as calpains, which are believed to play an important role in the breakdown of muscle structural proteins, and thus postmortem tenderization of meat [5,6]. Mutations in *CAST *resulting in unregulated calpain activity could therefore enhance meat tenderness. This notion has led to a number of studies relating *CAST *activity to meat tenderness [7,8].

In a recent study using Berkshire × Yorkshire (B × Y) F3 individuals with divergent meat quality phenotypes, sequencing of *CAST *coding regions as well as parts of the 5' and 3' untranslated regions (UTRs) revealed several single nucleotide polymorphisms (SNPs) [7]. SNP haplotypes were constructed and tested for association with a number of meat quality traits. One *CAST *haplotype was found to be associated with higher juiciness scores as well as lower firmness, Instron force and cooking loss scores. However, as each haplotype contained more than one SNP variant, no one mutation could be implicated as causative. Additionally, the possibility remains that the effects on meat quality are caused by an unidentified mutation in linkage disequilibrium with the observed polymorphisms.

Based on its known function and location, *CAST *could be considered a good candidate for the QTL influencing Instron shear force and sensory tenderness within the IMQP. However, for various reasons, it is unlikely that any of the *CAST *polymorphisms detected in the B × Y individuals [7] underlie the tenderness effects observed in our population. Firstly, the effects on tenderness observed in the B × Y population are not nearly as large as those in the IMQP; the additive effects calculated for shear force and tenderness in the IMQP are nearly two and three times the size of those for the B × Y family, respectively (when adjusted for differences in sensory tenderness scoring scales used) [1,9]. Secondly, in the IMQP, significance of QTL for these traits greatly exceeded the 1% genome-wise significance threshold, whereas the corresponding B × Y QTL did not reach the 5% chromosome-wise significance threshold. Thirdly, as mentioned, the reported IMQP QTL position is vague, i.e. not exclusive to *CAST*. Lastly, and perhaps most importantly, genotyping of the IMQP F_1 _boars for the known *CAST *SNPs revealed that two boars segregating for the QTL are not heterozygous for any of these SNPs. Therefore, the possibility remains that other polymorphisms, either in *CAST *or another gene, are responsible for the observed large effects.

Fine-mapping of QTL can be achieved by increasing marker density within the chromosomal region of interest, increasing the number of individuals for which phenotypic information can be obtained, or increasing the accuracy of assigning QTL genotype [10]. For this QTL, the most straightforward approach is to increase the number of informative markers within the QTL interval, as this alleviates the need to produce new animals or score new phenotypes on previously-generated animals. Recent advances in porcine genome resources, including the development of both a high-resolution bacterial artificial chromosome (BAC) fingerprint map [11,12] and a high-resolution whole genome radiation hybrid (WG-RH) map that integrates genetic, physical, and comparative mapping information [3], now provide reagents for the targeted isolation of new markers. Here we report the use of these resources to increase the marker density of the SSC2 linkage map, and thereby refine the map position of the IMQP pork tenderness QTL. Further refinement is suggested by haplotype analysis of the IMQP F_1 _boars, and together these data reinforce *CAST *as a strong positional candidate gene. Finally, data reported here also demonstrate the potential for additional QTL in this region influencing meat tenderness.

## Results

### Targeted marker selection and development

Based on the initial linkage analysis of SSC2, the most likely map position of the IMQP shear force and tenderness QTL coincides with that of microsatellite marker SW1517 [1]. To pinpoint the genomic locus of interest, SW1517 was positioned on the WG-RH comparative map [3]. Mapping of SW1517 indicated that it is centrally located within a large segment of conserved gene order with orthology to HSA5 between 72.39 and 150.11 Mb (NCBI build 36.2 coordinates). Specifically, this marker maps between BACs corresponding to human genomic positions of 104.48 and 106.04 Mb (see Additional file 1).  The *CAST *gene is also located within this large conserved segment, positioned between 96.08 and 96.14 Mb (based on BLASTn [13] results for porcine *CAST *sequence M20160).

The WG-RH comparative map was then referenced to identify publicly available microsatellite markers, as well as comparatively anchored BAC clones that could be used to target and develop additional markers within the ~30 Mb of genomic sequence (based on human coordinates; 89.38 to 118.74 Mb on HSA5) surrounding SW1517. Within this region are eight microsatellite markers and 23 mapped BAC end sequences (BESs; see Additional file 1). Of the eight microsatellite markers, six were included on the USDA/MARC linkage map; thus, marker information was readily available [2]. Polymorphism of these six markers was assessed within the IMQP, and four markers (SW766, SW1320, S0010 and SW1695) were found to be polymorphic in at least half of the F_1 _boars. These four markers were used for genotyping the entire IMQP. Eleven additional microsatellites, flanking the interval of interest or not mapped by RH, were selected from the USDA/MARC SSC2 linkage map; six markers (SW776, SW395, SW1628, S0370, SWR2157 and SW1879) were included in the genotyping and linkage analysis reported here, whereas five markers (SW1883, SW1860, SWR1512, SW1658 and SW1408) were discarded due to limited polymorphism within the IMQP population and/or suboptimal performance in genotyping assays.

BAC clones corresponding to the 23 WG-RH mapped BESs [3] surrounding SW1517 (see Additional file 1) were selected and used to generate two individual subclone libraries, each representing a pool of all clones. One of these libraries was enriched for microsatellite sequences. Additionally, a clone harboring the *CAST *gene (CH242-57F5) was selected, based on BAC fingerprint information and BES similarities to the human genome [11], and used to construct a third subclone library representing only this clone. Sequencing of 384 cloned inserts from the microsatellite-enriched library yielded 145 contigs, 58 (40%) of which contained simple sequence repeats (SSRs). From these 58 SSRs, 6 markers (EF444912, EF444913, EF444914, EF444915, EF444916 and EF444918) were developed and used for genotyping, as they each met four additional selection criteria; PCR primers could be designed to amplify the repeat, at least one primer could be designed in non-repetitive sequence, PCR amplification could be optimized for genotyping and polymorphism was observed in at least half of the F_1 _boars. Sequencing of ~0.5 Mb of the standard, i.e. non-enriched, pooled BAC library resulted in the identification of 45 novel SSRs. From these repeats, two markers (EF444911 and EF444917) were generated. Finally, partial sequencing of the *CAST*-containing BAC yielded 20 assembled contigs. From these 20 contigs, five SSRs were detected, and two additional markers (EF444909 and EF444910) were developed. In total, ten publicly available microsatellite markers and ten novel markers that represent seven different BACs were selected for genotyping the IMQP. The number of alleles per locus ranged from two to nine, with an average number of 4.7 alleles. A summary of marker data is provided in Table 1.

**Table 1:** Summary of marker data.

Marker^a^	Corresponding Porcine BAC Clone^b^	No. of Alleles	Min. Alllele Size (bp)	Max. Allele Size (bp)
SW776	--	3	104	116
SW395	--	3	143	163
SW766	--	3	151	162
EF444909	CH242-57F5	6	155	169
EF444910	CH242-57F5	2	175	182
EF444911	RP44-411O5	4	175	185
SW1320	--	3	126	161
EF444912	RP44-278B18	7	322	359
EF444913	RP44-278B18	6	286	302
SW1628	--	3	121	126
EF444914	RP44-280E2	9	134	165
EF444915	RP44-280F8	5	264	277
SW1695	--	6	174	188
EF444916	RP44-310M14	4	297	310
EF444917	RP44-310M14	3	336	357
S0370	--	6	137	157
SWR2157	--	6	107	129
SW1879	--	4	183	189
S0010^c^	--	5	93	122
EF444918^c^	RP44-426I5	5	224	238

^a^Genbank Accession or USDA/MARC microsatellite locus (S0 or SW) identifiers.

^b^RP44 and CH242 denote RPCI-44 and CHORI-242 Porcine BAC Library, respectively.

^c^Markers S0010 and EF444918 were omitted from linkage analyses due to a possible null allele and data incompatibility issues, respectively.

### Genotyping and linkage analysis

A total of 886 individuals of the IMQP, including 22 parental, 63 F_1_, and 801 F_2 _individuals were genotyped with 20 new markers. Using a LOD score threshold of 3.0, a multilocus linkage map including eighteen new, and five previously-mapped [1], microsatellite markers was constructed (Table 2). Two newly-genotyped markers were omitted from analyses due to the presence of a null allele and apparent data incompatibility that prevented map assembly. The order of markers was consistent with the WG-RH comparative map [3]. Total length of the sex-averaged map was 78.7 cM. Map distances between markers ranged from 0.0 to 18.8 cM, with mean and median marker separation of 3.6 and 1.1 cM, respectively. Within the chromosomal region of interest bounded by markers SW776 (21.5 cM) and SWR2157 (43.2 cM), mean and median marker separation distances were 1.3 and 0.4 cM, respectively.

**Table 2:** SSC2 linkage map.

Marker^a^	Map Distance (cM)	Map Position (cM)
SW1201^b^		0.0
	2.7	
SW1686^b^		2.7
	18.8	
SW776		21.5
	0.0	
SW395		21.5
	5.4	
SW766		26.9
	1.1	
EF444909		28.0
	0.0	
EF444910		28.0
	0.7	
EF444911		28.7
	0.3	
SW1320		29.0
	0.2	
EF444912		29.2
	0.0	
SW1517^b^		29.2
	0.0	
EF444913		29.2
	0.0	
SW1628		29.2
	1.3	
EF444914		30.5
	0.4	
EF444915		30.9
	4.2	
SW1695		35.1
	1.0	
EF444916		36.1
	0.2	
EF444917		36.3
	2.8	
S0370		39.1
	4.1	
SWR2157		43.2
	10.3	
SW1879		53.5
	10.4	
SW2192^b^		63.9
	14.8	
SWR308^b^		78.7

^a^Genbank Accession or USDA/MARC microsatellite locus (S0 or SW) identifiers.

^b^Denotes previously-mapped marker (Stearns et al. 2005).

**Figure 1:**
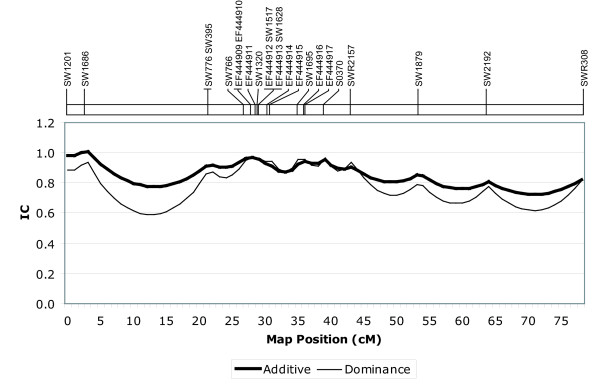
**Additive and dominance information contents calculated every 1 cM**. Relative cM position on the SSC2 linkage map is represented on the x-axis. Additive and dominance information contents, calculated according to the method of Knott et al. (1998), are plotted on the y-axis. The relative position of each mapped marker is indicated above the graph.

Information content (IC) was calculated at each centimorgan position of the linkage map [14,15] and values are plotted in Figure 1. Minimum additive and dominance information contents (IC) were 0.72 and 0.59, respectively; these minimum IC values were found within the two largest marker intervals, between the second and third markers, and the last two markers, as would be expected. Average additive and dominance ICs were 0.92 and 0.91, respectively, within the region of interest (SW776-SWR2157).

### QTL analyses

**Figure 2:**
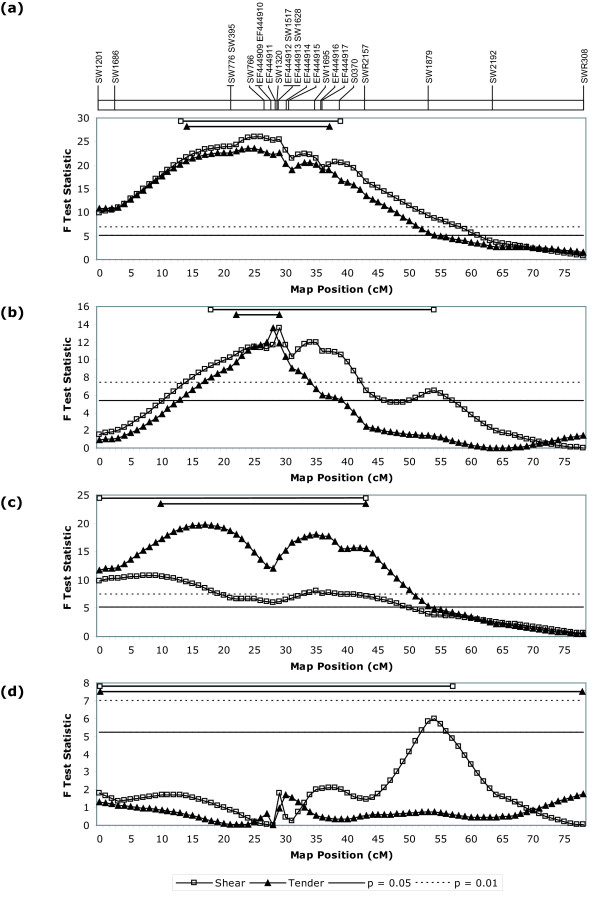
**F-test statistics for Instron shear force and taste panel tenderness calculated every 1 cM using a 1-QTL model**. Relative cM position on the SSC2 linkage map is represented on the x-axis, and calculated F-test statistics are represented on the y-axis. The 1% and 5% chromosome-wise significance thresholds are also indicated. The 95% confidence intervals for each trait are shown as horizontal lines at the top of each graph. Mapped marker positions and a comprehensive figure legend are provided above and below the set of graphs, respectively. Graphs show (a) all families of the IMQP, (b) combined families 108120 and 207061, (c) only family 108110, and (d) combined families 108120 and 207061 using a model that incorporated *CAST *markers as a fixed effect.

Phenotypic and marker data were analyzed, both across and within families, using the outbred F_2 _analysis servlet of QTL Express [14,16]. Both 1-QTL and 2-QTL models were utilized such that F-test statistics were computed for one-versus-zero QTL (Table 3) as well as two-versus-zero, and two-versus-one, QTL (Table 4). Using the 1-QTL model across all IMQP families, the most likely QTL positions for Instron shear force and sensory tenderness were concordant at 25 and 24 cM, respectively (Table 3, Figure 2a). Based on within-family analyses, this overall position primarily reflects the segregation of QTL alleles in the offspring of three IMQP F_1 _boars; families 108110, 108120 and 207061 yielded highly-significant F-test statistics for shear force and tenderness at 8 and 17 cM, 29 and 28 cM, and 22 and 28 cM, respectively (Table 3). F-statistics at these positions corresponded to chromosome-wise p-values < 0.01, with the exception of tenderness for family 207061 at 28 cM, which was significant at a level of p < 0.05. A combined analysis of families 108120 and 207061 revealed most likely QTL positions of 29 and 28 cM, both with p < 0.01, coinciding with the *CAST *markers positioned at 28 cM. Interestingly, family 207050 also demonstrated a most likely QTL position of 28 cM for shear force, although not at a p < 0.05 significance level; the F-test statistic for this position was 4.31, whereas the p = 0.05 significance threshold was 5.48.

**Table 3:** Outbred F_2_ QTL analyses using a 1-QTL model.

F_1_ Boar^a^	No. of Full-Sib Families	Total No. of Offspring	Trait^b^	QTL Position (cM)	F-Test Statistic^c^	Likelihood Ratio	LOD	Additive Effect	S.E.^d^	Dominance Effect	S.E.^e^	95% C.I. of QTL Position^f ^(cM)	Length of C.I. (cM)
IMQP	86	801	Shear	25	**26.07	50.47	10.96	-0.21	0.03	-0.05	0.04	13.0 – 39.0	26.0
IMQP	86	801	Tender	24	**23.57	45.78	9.94	0.57	0.08	0.06	0.12	14.0 – 37.0	23.0
108110	13	141	Shear	8	**10.75	19.85	4.31	-0.43	0.10	-0.24	0.16	0.0 – 43.0	43.0
108110	13	141	Tender	17	**19.70	34.29	7.45	1.31	0.22	0.40	0.32	10.0 – 43.0	33.0
108120	16	158	Shear	29	**8.22	15.59	3.39	-0.24	0.06	-0.06	0.09	21.5 – 77.0	55.5
108120	16	158	Tender	28	**10.40	19.46	4.23	0.72	0.17	0.39	0.24	22.0 – 38.0	16.0
207050	18	126	Shear	28	4.31	8.31	1.81	-0.17	0.06	-0.04	0.08	0.0 – 78.0	78.0
207050	18	126	Tender	3	4.13	7.98	1.73	0.48	0.21	-0.61	0.30	0.0 – 77.5	77.5
207061	10	110	Shear	22	**10.51	19.03	4.13	-0.26	0.07	-0.23	0.11	14.5 – 46.0	31.5
207061	10	110	Tender	28	*6.18	11.64	2.53	0.63	0.18	-0.09	0.28	7.5 – 76.0	68.5
308102	11	105	Shear	44	0.98	1.94	0.42	-0.01	0.08	-0.16	0.11	0.0 – 78.0	78.0
308102	11	105	Tender	47	1.74	3.41	0.74	0.12	0.27	0.68	0.37	0.0 – 75.0	75.0
309050	17	148	Shear	23	4.59	8.89	1.93	-0.16	0.05	-0.01	0.08	1.0 – 78.0	77.0
309050	17	148	Tender	33	4.93	9.51	2.07	0.50	0.18	0.44	0.27	0.0 – 53.5	53.5
108120, 207061	26	268	Shear	29	**13.54	25.72	5.59	-0.22	0.05	-0.12	0.07	18.0 – 54.0	36.0
108120, 207061	26	268	Tender	28	**13.53	25.71	5.58	0.64	0.13	0.18	0.19	22.0 – 29.0	7.0
108120, 207061^g^	26	268	Shear	54	*5.97	11.66	2.53	0.03	0.05	0.26	0.07	0.0 – 57.0	57.0
108120, 207061^g^	26	268	Tender	78	1.74	3.45	0.75	-0.05	0.14	0.35	0.19	0.0 – 78.0	78.0

^a^IMQP indicates all animals of the Illinois Meat Quality Pedigree analyzed in this study.

^b^Shear and Tender denote Instron Shear Force and Taste Panel Tenderness, respectively.

^c^Values significant at the p = 0.05 and p = 0.01 levels, as determined by chromosome-wise permutation (*n *= 5,000), are indicated with one and two asterisks, respectively.

^d^Denotes standard error associated with additive effects.

^e^Denotes standard error associated with dominance effects.

^f^Confidence interval as determined by bootstrapping (*n *= 1,000).

^g^Includes *CAST *as a fixed genetic effect.

**Table 4:** Outbred F_2_ QTL analyses using a 2-QTL model.

F_1_ Boar^a^	Trait^b^	QTL Position A (cM)	QTL Position B (cM)	2 vs. 0 QTL F-Test Statistic	2 vs. 1 QTL F-Test Statistic	Likelihood Ratio	LOD	Additive Effect QTL A	S.E.^c ^QTL A	Dominance Effect QTL A	S.E.^d ^QTL A	Additive Effect QTL B	S.E.^c^ QTL B	Dominance Effect QTL B	S.E.^d ^QTL B
IMQP	Shear	25	54	14.42	2.65	55.70	12.10	-0.20	0.03	-0.07	0.05	-0.02	0.04	0.10	0.05
IMQP	Tender	24	35	14.42	5.13	55.92	12.14	0.56	0.14	-0.26	0.16	0.03	0.14	0.47	0.15
108110	Shear	2	43	6.32	1.76	23.20	5.04	-0.31	0.10	-0.21	0.14	-0.21	0.11	0.11	0.14
108110	Tender	15	43	10.70	1.53	37.07	8.05	0.94	0.30	0.27	0.37	0.48	0.27	0.14	0.31
108120	Shear	34	54	5.71	2.98	21.37	4.64	-0.28	0.08	-0.10	0.10	0.11	0.07	0.23	0.11
108120	Tender	1	28	6.11	1.72	22.75	4.94	-0.29	0.21	-0.37	0.28	0.90	0.20	0.51	0.26
207061	Shear	29	39	7.18	3.34	25.36	5.51	0.09	0.13	-0.60	0.16	-0.33	0.13	0.40	0.16
207061	Tender	18	23	4.35	2.35	16.16	3.51	-2.16	1.17	2.82	1.32	2.73	1.12	-2.70	1.24
108120, 207061	Shear	40	53	11.07	7.85	40.92	8.89	-0.33	0.07	-0.20	0.08	0.19	0.07	0.34	0.09
108120, 207061	Tender	28	78	7.67	1.74	29.07	6.31	0.64	0.13	0.18	0.19	-0.05	0.14	0.35	0.19

^a^IMQP indicates all animals of the Illinois Meat Quality Pedigree analyzed in this study.

^b^Shear and Tender denote Instron Shear Force and Taste Panel Tenderness, respectively.

^c^Denotes standard error associated with additive effects.

^d^Denotes standard error associated with dominance effects.

Families exhibiting significant effects using a 1-QTL model were further analyzed using a 2-QTL model (Table 4). Although significance thresholds could not be computed using this model in QTL Express, these analyses suggested the possibility of more than one QTL for both shear force and tenderness traits on SSC2. F-test statistics calculated with the two-versus-one QTL model exceeded 5.0 for the overall IMQP analysis, yielding sensory tenderness QTL positions at 24 and 35 cM. Likewise, the same analysis, using the combined dataset for families 108120 and 207061, resulted in an F-statistic of 7.85 for shear force QTL at positions at 40 and 53 cM.

### Haplotype analysis of F_1 _boars

**Figure 3:**
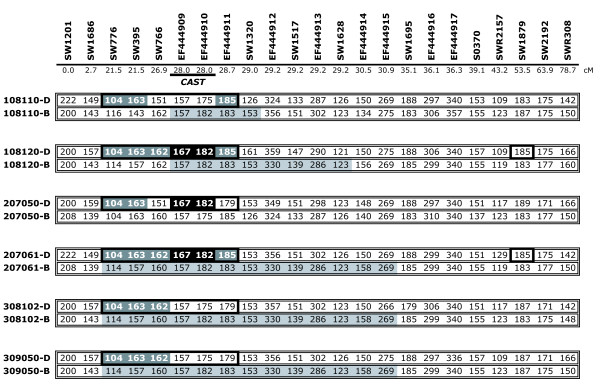
**Haplotype analysis of the IMQP F_1 _boars**. Each chromosomal segment is depicted as a series of marker alleles, designated by allele size and ordered relative to the linkage map. Marker names and map positions, in cM, are indicated above the respective alleles. Individual boar IDs, as well as the breed of origin (B = Berkshire, D = Duroc), are indicated to the left of each segment. Black boxes indicate haplotypes shared by both individuals (108120, 207061) that appear to be segregating for QTL at the *CAST *position as well as a putative secondary position near marker SW1879. Duroc alleles shaded in dark gray are also shared with non-segregating boars, and only those alleles shaded in black are unique to individuals with coincident QTL positions. Berkshire alleles shaded in light gray indicate shared alleles in the chromosomal region of interest.

By comparing genotypic data between the IMQP F_1 _boars and their respective parents, parental linkage phase was determined for all markers; deduced maternal (Duroc) and paternal (Berkshire) chromosomal segments inherited by each of the six F_1 _boars are shown in Figure 3. Berkshire chromosomal segments appeared highly similar between markers SW776 and S0370 for both segregating (108120, 207061) and non-segregating (308102, 309050) boars; only the 108120 alleles for SW766 and EF444914 differed from the other three individuals in this region. In the region surrounding the most likely QTL position, five of six F_1 _boars inherited four shared alleles from their respective sires, suggesting that these paternal alleles do not underlie the variation observed in the IMQP.  Comparison of the Duroc chromosomal segments revealed a haplotype shared by both individuals segregating for QTL in the 28.0–29.0 region (108120, 207061); this haplotype spans the region between markers SW776 and EF444911 (21.5–28.7 cM). However, alleles of the first three markers in this region (SW776, SW395 and SW766) do not appear to be associated with the QTL, as they are common among segregating and non-segregating F_1 _boars; in fact, the first two marker alleles are shared by all six boars. Likewise, the last marker allele of this haplotype is shared with boar 108110; although this boar also appears to be segregating for these traits, the most likely QTL positions do not coincide with those of 108120 and 207061 (Table 3, Figure 3). The remaining two marker alleles of the haplotype, centered at *CAST*, are shared with boar 207050; although not statistically-significant in this family, the most likely QTL position for shear force coincides with these markers.

## Discussion

By taking advantage of recent developments in porcine genome resources, including high-resolution WG-RH comparative and BAC fingerprint maps, we have fine-mapped a recently-reported QTL exhibiting large effects on both Instron shear force and sensory tenderness phenotypes in a Berkshire × Duroc pedigree. First, we utilized the radiation hybrid technique to incorporate microsatellite marker SW1517 into the WG-RH comparative map; as the position of this marker coincided with the most likely position of the tenderness QTL, we were able to define the locus of interest. Additionally, as the majority of the markers comprising the RH map are physically anchored, i.e. derived from BAC clones, SW1517 could be generally positioned on the BAC physical map, thereby defining the BAC resources available for the targeted isolation of new informative markers. By utilizing the BAC resources in the ~30 Mb surrounding the locus of interest, we were able to increase local marker density on the SSC2 linkage map by addition of eighteen markers and refine the map position of the tenderness QTL through linkage and haplotype analyses.

Outbred F_2 _analysis of all IMQP genotypic and phenotypic data, using a 1-QTL model, positioned QTL for Instron shear force and taste panel tenderness proximal to SW766, at 25 and 24 cM, respectively (Table 3). In our population, this location represents segregation of QTL alleles within three different F_1 _boar families, and may be skewed as a result of differing QTL positions; one boar displayed most likely QTL positions located 11–21 cM proximal to those of the other two boars found at 28–29 cM. These findings seem to be in agreement with a recently-conducted genome scan for loci influencing pork quality traits in a Duroc × Landrace F_2 _population [17]. A number of associations were reported within the SSC2 region between markers SW1026 and SW1370; these included a QTL of genome-wise significance for taste panel overall tenderness as well as a suggestive QTL for slice shear force at day seven postmortem. Based on available map information, this QTL region also corresponds to the segment of SSC2q proximal to marker SW766 (Table 2); SW1026 has previously been mapped to the centromeric end of SSC2p [2-4] and SW1370 was previously localized to the same position as marker SW766 [4]. However, the four F_1 _boars of the Duroc-Landrace population were not analyzed individually; in light of our data, it may prove interesting to investigate whether there are differing QTL positions associated with the boars in that population.

Based on the direction of effects observed in the QTL analyses, the Berkshire allele appears to be associated with increased taste panel tenderness and decreased shear force (Table 3). As the Duroc breed has been ranked lower than the Berkshire with respect to meat quality traits such as tenderness [18,19], this might be expected. However, haplotype analysis of the six F_1 _boars of the IMQP revealed a common haplotype, corresponding with the most likely QTL positions, among the maternal (Duroc) chromosomes (Figure 3). Additionally, if it is assumed that shared alleles within the refined interval represent segments that are identical-by-descent, the high similarity observed among paternal (Berkshire) chromosomes of both segregating and non-segregating boars also implies that any variation underlying the phenotypes observed in the IMQP was likely inherited from the dams. These findings suggest that the QTL detected within the IMQP more likely represents a Duroc allele promoting meat toughness rather than a Berkshire allele enhancing meat tenderness. This notion is also supported by the observation in the Duroc-Landrace population that the Duroc allele decreased taste panel tenderness and increased shear force at day seven postmortem [17].

Based on a number of studies relating CAST activity to meat tenderness in other animals, the calpastatin gene is considered a good positional candidate for underlying the phenotypic effects observed in the IMQP. However, for reasons mentioned previously, involvement of other genes and/or molecular variation could not be excluded, warranting refinement of the QTL interval. The fine-mapping data presented here reinforces *CAST *as a strong positional candidate. Firstly, RH mapping of SW1517 localized this marker to the center of a large comparative segment of conserved gene order on SSC2q with orthology to the region of HSA5 harboring *CAST *(see Additional file 1). Secondly, the development of markers from a *CAST*-containing BAC and the inclusion of these markers in linkage analyses revealed a strong positional agreement between these markers and the most likely QTL positions for one or both tenderness traits in two, and perhaps three, individual families (Table 3); statistical support was only evident for families 108120 and 207061, although a shear force QTL was nearly significant at the same position in family 207050. Furthermore, a combined analysis including both statistically-significant families reduced the 95% confidence intervals for shear force and tenderness from essentially the entire q arm of SSC2 to 36 and 7 cM, respectively, with the most likely position centered at the *CAST *markers (Table 3, Figure 2b). Finally, haplotype analysis of the F_1 _boars suggests further refinement of the interval as the common maternal haplotype among heterozygous boars and nearest the most likely QTL position is comprised of only the two markers derived from the *CAST*-containing BAC (Figure 3). Analysis of the paternal chromosomes distinguishes boar 207050 from the other similar individuals, perhaps revealing differences in background effects that could account for the difference in significance observed between this individual and the other two individuals sharing the common maternal haplotype; alternatively, this difference in significance may suggest the potential for multiple *CAST *alleles, with varying degrees of influence on pork tenderness.

Although fine-mapping of the IMQP tenderness QTL strengthens support for *CAST *as a positional candidate, little is known about the molecular variation among *CAST *alleles within this population. Determination of the entire *CAST *genomic sequence and re-sequencing of this gene from DNA of the six main IMQP F_1 _boars should provide further insight into the molecular basis of the pork tenderness effects observed in this population.

Besides providing justification for detailed characterization of *CAST *variation within the IMQP, data presented here also indicate that additional tenderness QTL may exist on SSC2q; map positions for any additional QTL, however, remain ambiguous. Unexpectedly, QTL analysis of the 108110 family revealed highly-significant (p < 0.01) QTL for shear force and tenderness positioned proximal to *CAST*, at 8 and 17 cM, respectively (Table 3). As these positions are in a region of relatively low information content (Figure 1), due to a lack of markers between 2.7 and 21.5 cM, it is unclear whether this variance arises from one, or more than one, map position. Apparently, the significance observed in this region cannot be attributed to the *CAST *position at 28 cM, as inclusion of this position as a background genetic effect in the QTL model yields the same QTL, albeit with reduced significance (p < 0.05; data not shown). It is also interesting to observe that, for family 108110, a considerable drop in F-test statistic coincides with the *CAST *position (Figure 2c); this further suggests that the *CAST *locus is not responsible for the differences in tenderness phenotypes observed in this particular family and that additional QTL may exist on SSC2q.

In addition to the 1-QTL analysis of 108110 offspring supporting a QTL position proximal to *CAST*, the 2-QTL analyses of the complete IMQP, as well as the combined 108120 and 207061 families, may suggest the presence of QTL distal to *CAST *(Table 4). Results of the overall IMQP analysis using a 2-QTL model show a secondary QTL position for taste panel tenderness at 35 cM, corresponding to the location of marker SW1695 (35.1 cM); interestingly, the interval between this marker and marker S0370 was found to contain a suggestive QTL for shear force at day two postmortem in the Duroc-Landrace genome scan [17]. Results of the combined analysis of families 108120 and 207061 using a 2-QTL model unexpectedly seemed to support QTL for shear force, both located distal to *CAST*, with most likely positions at 40 and 53 cM; the presence of two-versus-one QTL was supported by an F-test statistic of 7.85 (Table 4). Although analysis of the same dataset, using the 1-QTL model, provides the strongest support for a QTL at the *CAST *position and affirms this gene as a strong positional candidate, a secondary QTL position is evident at 54 cM (Figure 2b). Inclusion of *CAST *as a fixed genetic effect in this model does not affect the location or significance (p < 0.05) of this secondary position (Figure 2d). Given the results of the 1-QTL analysis, it is unclear why the 2-QTL model does not position QTL at 28 cM (*CAST*) and 54 cM. The potential for shear force QTL positioned both at, and distal to, *CAST *may explain why the 95% confidence interval (C.I.) for this trait, based on the 1-QTL analysis of these combined families, could not be reduced beyond 36 cM, whereas the C.I. for tenderness was reduced to 7 cM; 2-QTL analysis of this combined dataset shows no significant support for a taste panel tenderness QTL distal to *CAST*. One might expect that this putative second QTL would affect both traits similarly. However, the phenotypic correlation between mechanical shear force and taste panel tenderness is only estimated to be -0.66 [20], thus allowing for the detection of QTL for one trait independent of the other.

By targeting SSC2q for marker isolation, additional resources have been generated that could aid in new investigation of other QTL on this chromosome arm, or further refinement of the QTL interval positioned at *CAST*. From the pooled BAC library constructed in this study, an additional ~0.75 Mb of sequence information has been generated, and can be used for the isolation of new informative microsatellite or SNP markers. Furthermore, the use of physically-anchored BAC clones allowed for the selection of a minimal tiling path (MTP) of clones spanning the ~30-Mb region of interest interrogated here; from the fingerprinted contig #2008, 224 clones, or approximately eight clones per Mb, including the 23 clones of the pooled BAC library, were selected. This MTP can now be referenced to develop markers in remaining regions of relatively low marker density for further refinement of QTL positions.

## Conclusion

Through the use of a targeted approach to new marker development exploiting recently-developed high-resolution porcine genome maps, we have nearly tripled the marker density of the SSC2 linkage map, and thereby fine-mapped a QTL with large effects on pork tenderness within the IMQP resource population. Refinement of this QTL interval strengthened support for the positional candidate, calpastatin, and suggested that the observed effects may be the result of a Duroc-derived allele that decreases tenderness.  Additionally, other putative QTL were suggested in this chromosomal region. Future studies directed toward the molecular characterization of *CAST *alleles present in the IMQP, as well as further investigation of other putative QTL revealed in this study, should increase current knowledge of the genetic factors influencing pork tenderness and thus improve the quality of pork products for consumers. The targeted approach to interval refinement employed here should prove applicable to positional cloning efforts for other QTL of importance to the swine industry.

## Methods

### Resource population and phenotypic data

The Illinois Meat Quality Pedigree (IMQP), generated from a Berkshire × Duroc intercross, has been generally described [1]. This pedigree represents a three-generation resource population in which each of three purebred Berkshire boars was mated to six or seven different purebred Duroc sows (*n *= 19) to produce seven F_1 _boars and fifty-six F_1 _sows. Non-sibling F_1 _individuals were then intermated. Six of the seven F_1 _boars were primarily selected for matings such that two paternal half-sib boars sired by each of three founder Berkshire sires were used to generate F_2 _individuals; these boars were named 108110, 108120, 207050, 207061, 308102 and 309050, where the first number indicates the founder sire of each F_1 _boar. The seventh boar, 207020, sired only one family of thirteen offspring and was therefore not analyzed individually in this study. Only F_2 _pigs for which shear force and sensory tenderness data were available were used. These F_2 _pigs included 801 individuals from eighty-six full-sib families; the number of full-sib families and the total number of offspring sired by each F_1 _boar are indicated in Table 3. Individual full-sib families represented up to three litters and ranged in size from two to twenty-four pigs, with an average family size of approximately nine pigs.

The phenotypic data used in this study have previously been reported, and the appropriate methods of collection have been described [1]; briefly, shear force was measured using a Universal Testing Machine (Instron) with a Warner-Bratzler shear attachment, and sensory tenderness was scored, using an integer scale of 1 (tough) to 15 (tender), by a trained panel of six independent testers.

### RH mapping of microsatellite marker SW1517

Primer sequences for microsatellite marker SW1517 were obtained from the United States Department of Agriculture/Roman L. Hruska Meat Animal Research Center (USDA/MARC) [2]. This marker was then amplified by PCR, using INRA-Minnesota porcine radiation hybrid (IMpRH) panel DNA templates, as described for previously mapped markers [3]. SW1517 vector data was then added to existing SSC2 marker vector data and used to construct a multipoint maximum likelihood RH map as described.

### Selection and screening of publicly available markers

Primer sequences for microsatellite markers previously mapped to SSC2q were obtained from the USDA/MARC website [2]. PCR was typically performed in a 10-μl reaction volume containing 20–25 ng of template DNA, 1× PCR buffer (containing 1.5 mM MgCl_2_; QIAGEN), 200 μM each dNTP (Fermentas), 0.5 μM each primer, and 0.25 U HotStarTaq DNA polymerase (QIAGEN). Typical PCR cycling parameters included an initial denaturation step of 95°C for 15 min followed by 35 cycles of 94°C for 30 s, 55–66°C for 45 s, and 72°C for 45 s, plus a final extension step of 72°C for 5 min. One primer of each pair was radioactively end-labeled, using an appropriate reaction volume containing 1× T4 polynucleotide kinase (PNK) buffer (New England Biolabs), 10 μM primer, 5 U T4 PNK, and 0.3 μCi γ-^32^P dATP.  Labeling was performed for 30 min at 37°C, followed by an enzyme inactivation step of 65°C for 10 min. Radioactively-labeled primers were then used to PCR-amplify each marker from genomic DNA of the six F_1 _boars. Length polymorphism was assessed by polyacrylamide gel electrophoresis. Markers with at least three alleles and determined to be polymorphic in at least three of six F_1 _boars were used for genotyping all individuals in the IMQP.

### Development of novel informative markers

#### Construction of standard BAC subclone libraries

Twenty-three anchored BAC clones, as well as any potential *CAST*-containing clones, were obtained from the appropriate BAC libraries (CHORI-242 or RPCI-44) [21,22]. BAC clones were individually cultured overnight in 3 ml 2×LB media containing 20 μg/ml chloramphenicol, at 37°C with shaking. Aliquots of these cultures were then used to inoculate one of two 100-ml cultures (2×LB media, 20 μg/ml chloramphenicol). For the pooled BAC library, 100 μl of each appropriate culture (2.3 ml total) was used for inoculation, and for the *CAST*-containing BAC library, 2.5 ml of the one appropriate culture was used. Both cultures were incubated at 37°C, with shaking, for an additional 6.75 hrs, until an OD_600 _of ~2.0 was attained. Cultures were then centrifuged for 15 min at 3,000 × *g*, and BAC DNA was isolated using the NucleoBondR^® ^BAC 100 protocol (Macherey-Nagel). STS content was confirmed by PCR, using purified BAC DNA as template, for all 23 BACs in the BAC pool, as well as for the *CAST*-containing BAC.

Subclone libraries were generated using the TOPO^® ^Shotgun Subcloning Kit (Invitrogen). For each library, ~5 μg of purified BAC DNA was sheared, by nebulization, to obtain DNA fragments with a median size of ~2 kb (pooled BAC library) or ~900 bp (*CAST*-containing BAC library). As an optional step in the protocol, sheared DNA was size-fractionated and purified before proceeding; SizeSep™ 400 Spun Columns (Amersham Pharmacia Biotech) were used, according to instructions and using 1× NEBuffer2 (New England Biolabs; 10 mM Tris-HCl, 50 mM NaCl, 10 mM MgCl_2_, 1 mM Dithiothreitol, pH 7.9) for column equilibration, to remove any small molecules and DNA fragments < 400 bp in size. Subsequent DNA modification and cloning steps were done according to the manufacturer's protocol.

#### Construction of a microsatellite-enriched BAC subclone library

As described above, ~5 μg of the same purified BAC DNA used to construct the standard pooled BAC subclone library was nebulized to obtain DNA fragments with a median size of ~2 kb. Sheared DNA was then size-fractionated/purified and the DNA ends were blunted, also as described.

DNA linkers were prepared, by mixing equal Molar volumes of two HPLC-purified oligonucleotides, cDNA-1b (5'-GTCACGCAAGCTTCTCACAGG-3') and cDNA-2b (5'phos-CCTGTGAGAAGCTTGCGTGACTT-3'), boiling for 5 min, and slowly cooling to room temperature. Linkers were then ligated to the blunt-ended DNA fragments in a 100-μl reaction volume of 1× T4 DNA ligase buffer (New England Biolabs; 50 mM Tris-HCl, 10 mM MgCl_2_, 1 mM ATP, 10 mM Dithiothreitol, 25 μg/ml BSA, pH 7.5) containing approximately a 1:10 ratio of fragment ends:linker (~3 pmol ends:~30 pmol linker) and an excess (2,000 U) of T4 DNA ligase. Ligation was allowed to proceed for 2 hrs at room temperature.

Ten microliters of the ligation reaction was then used as template in a 200-μl PCR reaction containing 1× PCR buffer (containing 1.5 mM MgCl_2_; QIAGEN), 200 μM each dNTP (Fermentas), 1 μM non-HPLC purified cDNA-1b primer, and 5 U Taq DNA polymerase (QIAGEN). PCR cycling parameters included initial elongation and denaturation steps of 63°C for 10 min and 95°C for 3 min, respectively, followed by 26 cycles of 94°C for 1 min, 64°C for 1 min, and 72°C for 2.5 min, plus a final extension step of 72°C for 5 min. Ten micrograms of library PCR product DNA was then purified using the QIAquick PCR purification kit, according to the protocol, and eluted in 40 μl Buffer EB (10 mM Tris-Cl, pH 8.5).

Purified library DNA was then enriched twice for CA_n _microsatellites using a 5'-biotinylated CA_15 _oligonucleotide probe and streptavidin-coated beads (Dynal). For the first enrichment, 0.5 μg of library DNA was hybridized with 10 μM biotinylated CA_15 _oligonucleotide in a 10-μl reaction volume of 1× hybridization buffer [1.5 M NaCl, 10 mM NaHPO_4 _(pH 7.2), 10 mM EDTA, 10× Denhardt's Solution (0.2% Ficoll, 0.2% polyvinylpyrolidone, 0.2% BSA), 0.2% SDS]. Hybridization was allowed to proceed overnight, at 72°C, following an initial denaturation step of 95°C for 10 min. The hybridization reaction was then placed on ice for 3 min, before adding 50 μl of Bead Binding Buffer (BBB; 10 mM Tris (pH 7.5), 1 mM EDTA, 1 M NaCl), and transferring the entire volume to a microcentrifuge tube containing 100 μl of pre-washed streptavidin-coated beads in BBB (washed twice with 1 ml BBB plus 1× BSA, then once with 1 ml BBB). Hybridization of DNA to beads was allowed to proceed for 25 min at room temperature, followed by three 15-min washes with 1 ml of pre-warmed wash solution at 72°C (1× SSC, 0.1% SDS). DNA was then eluted from the beads by addition of 50 μl of 50 mM NaOH for 5 min, transferred to a new tube and neutralized with 50 μl 1 M Tris (pH 7.5). DNA fragments were then purified using the QIAquick PCR purification kit and eluted in 50 μl Buffer EB.

Ten microliters of purified, enriched library DNA was then used as template in a 200-μl PCR reaction containing 1× PCR buffer (containing 1.5 mM MgCl_2_; QIAGEN), 200 μM each dNTP (Fermentas), 1 μM non-HPLC purified cDNA-1b primer, and 5 U HotStarTaq DNA polymerase (QIAGEN). PCR cycling parameters included an initial denaturation step of 95°C for 15 min, followed by 30 cycles of 94°C for 1 min, 64°C for 1 min, and 72°C for 2.5 min, plus a final extension step of 72°C for 5 min. Products were again purified using the QIAquick PCR purification kit and eluted in 40 μl sterile Optima water (Fisher Scientific).

The second enrichment involved an additional hybridization reaction, containing 30 μl of purified, enriched PCR product in 1× hybridization buffer with ~1.6 μM biotinylated CA_15 _oligonucleotide. Hybridization proceeded for ~3 hrs, following an initial denaturation step of 95°C for 10 min. Hybridization was then stopped, beads were washed, and DNA was eluted as described above. PCR was performed, as above, in a 100-μl reaction volume. One microliter of double-enriched PCR product was then for ligation and cloning, according to the protocol for the TOPO^® ^TA cloning kit for sequencing (Invitrogen, pCR^®^4-TOPO^® ^cloning vector).

#### Skim sequencing of subclone libraries

Library transformants were randomly picked and grown overnight, at 37°C with shaking, in 96-well culture plates containing 1.2–1.5 ml 2× LB media plus 100 μg/ml ampicillin per well. Plasmid DNA was isolated using a standard alkaline lysis protocol, and 100–200 ng of each plasmid was used as template for cycle sequencing. Sequencing was performed in an 8-μl reaction volume containing ~1.3 μM primer T3 (5'-ATGACCATGATTACGCCAAGC-3') or T7 (5'-ATACGACTCACTATAGGGCGAA-3'), 0.25 μl Big Dye v3.1, 0.08 μl Big Dye dGTP v3.0, and 3.62 μl dilution buffer (0.16 M Tris base (pH 9.0), 3 mM MgCl_2_, 4.9% tetramethylene sulfone, 0.0001% Tween-20^® ^surfactant) [23]. Cycle sequencing parameters included an initial denaturation step of 96°C for 1.5 min followed by 45 cycles of 96°C for 15 s, 53°C for 15 s, and 60°C for 3 min, plus a final extension step of 60°C for 10 min. Sequencing products were then purified by size exclusion using Sephadex^® ^G-50 Fine (Amersham Biosciences) and run on an ABI 3730 capillary sequencer (Applied Biosystems). Sequences were analyzed using Phred base-calling and Phrap assembly software [24]; sequences were trimmed of low-quality reads (Phred quality score < 20) as well as vector sequence prior to assembly. Any contaminating *E. coli *sequence, as identified by BLASTn, was removed, and assembled contigs or individual reads were screened for simple repeats (with a minimum length of 8 per dimer, 6 per trimer, and 6 per tetramer repeat) using the online Simple Sequence Repeat Identification Tool (SSRIT) [25]. Sequence contigs or reads containing SSRs were then masked of additional porcine repetitive elements using RepeatMasker [26] prior to primer design. Primers were designed using available tools including Primer Designer 2 (Scientific and Educational Software) and Primer 3 [27].

#### Markers

Markers developed in this study were named according to assigned GenBank accession numbers [GenBank: EF444909, EF444910, EF444911, EF444912, EF444913, EF444914, EF444915, EF444916, EF444917, EF444918].

### Genotyping and linkage analysis

One primer of each pair per selected marker was labeled with one of four fluorescent dyes (PET™, NED™, VIC^®^, 6-FAM™; Applied Biosystems), and grouped into multiplex PCR reactions based on color and size combinations. PCR conditions were optimized accordingly, resulting in five multiplexes of two to six markers. IMQP DNA templates (including "no DNA" controls) were prepared in 10.5 96-well PCR plates, and PCR was performed using 0.4–1 μM primer, and either QIAGEN Multiplex PCR Master Mix or HotStarTaq, with appropriate buffers. Multiplex PCR conditions are described in Additional file 2.

PCR products from three and two multiplexes, respectively, were combined and purified using Promega Wizard^® ^SV96 binding plates. Briefly, 5 μl of each multiplex PCR product was combined, with or without water, to a final volume of 15 μl. Combined products were then mixed with 75 μl of isopropanol, and transferred to a binding plate. Following binding for ~1 min, liquid was removed by vacuum filtration. Bound products were then washed three times with 200 μl of 80% ethanol and eluted in 80 μl Optima water. GeneScan™ 500 LIZ^® ^size standard (Applied Biosystems) was added to 10-μl aliquots prior to loading on an ABI 3730 capillary sequencer. Automated allele-calling was performed using either GeneMapper^® ^v4.0 (Applied Biosystems) or GeneMarker^® ^(SoftGenetics, LLC) software. Allele calls were checked manually and edited if necessary.

A multilocus linkage map was constructed using CRI-MAP v2.4 [28]. The TWOPOINT option was used to calculate two-point linkage between marker pairs, and markers displaying a recombination fraction of 0.0 were haplotyped. The BUILD option was then used to map markers, in decreasing order of informativeness, with a LOD score threshold of 3.0. The CHROMPIC option was used to identify and remove potential genotype errors.

### QTL analyses

Phenotypic and marker data were analyzed, both across and within families, using the outbred F_2 _analysis servlet of QTL Express [14,16]. QTL, additive and dominance effects were estimated every 1 cM for both Instron shear force and taste panel tenderness traits, using a general linear model including an overall trait mean, additive and dominance effects, fixed effects, and a residual error term. Fixed effects included sex (2 levels) and birth month and year (BYM; 14 levels). Chromosome-wise significance thresholds were determined by permutation (*n *= 5,000), and 95% confidence intervals were obtained by bootstrapping (*n *= 1,000). Both 1- and 2-QTL models were used; however, chromosome-wise significance thresholds could not be determined for a 2-QTL model using QTL Express.

## Authors' contributions

SNM performed RH mapping, selected and screened available microsatellite markers, selected BACs, constructed BAC subclone libraries, sequenced subclones, isolated and developed novel microsatellite markers, optimized and performed genotyping reactions, performed manual editing of allele calls and genotypes, constructed the linkage map, performed QTL and haplotype analyses and drafted the manuscript. SLRZ provided previously-published genotypic and phenotypic data, participated in the design of the study and contributed to the interpretation of data. JEB also provided previously-published genotypic and phenotypic data, performed sequence analysis and assembly, aided in primer design, supervised the research, contributed to the design of the study as well as the interpretation of results, and assisted in drafting the manuscript. All authors have read and approved the final manuscript.

## Supplementary Material

Additional file 1 Local RH map including SW1517. This table demonstrates the incorporation of microsatellite marker SW1517 into the existing human-pig comparative radiation hybrid map [3]. The map position of SW1517 is shown relative to other markers within the chromosomal (SSC2) region of interest. Click here for file

Additional file 2 Multiplex PCR genotyping conditions. This table provides PCR primer sequence information as well as multiplex PCR reaction conditions used to genotype each microsatellite marker. Click here for file
